# Mechanisms and evolution of resistance to environmental extremes in animals

**DOI:** 10.1186/s13227-019-0143-4

**Published:** 2019-11-18

**Authors:** Thomas C. Boothby

**Affiliations:** Department of Molecular Biology, University of Wyoming, Laramie, WY USA

**Keywords:** Stress tolerance, Freeze tolerance, Anhydrobiosis, Thermotolerance, Radiotolerance, Evolution of stress tolerance, Cross tolerance

## Abstract

When animals are exposed to an extreme environmental stress, one of three possible outcomes takes place: the animal dies, the animal avoids the environmental stress and survives, or the animal tolerates the environmental stress and survives. This review is concerned with the third possibility, and will look at mechanisms that rare animals use to survive extreme environmental stresses including freezing, desiccation, intense heat, irradiation, and low-oxygen conditions (hypoxia). In addition, an increasing understanding of the molecular mechanisms involved in environmental stress tolerance allows us to speculate on how these tolerances arose. Uncovering the mechanisms of extreme environmental stress tolerance and how they evolve has broad implications for our understanding of the evolution of early life on this planet, colonization of new environments, and the search for novel forms of life both on Earth and elsewhere, as well as a number of agricultural and health-related applications.

## Introduction

**Fig. 1:**
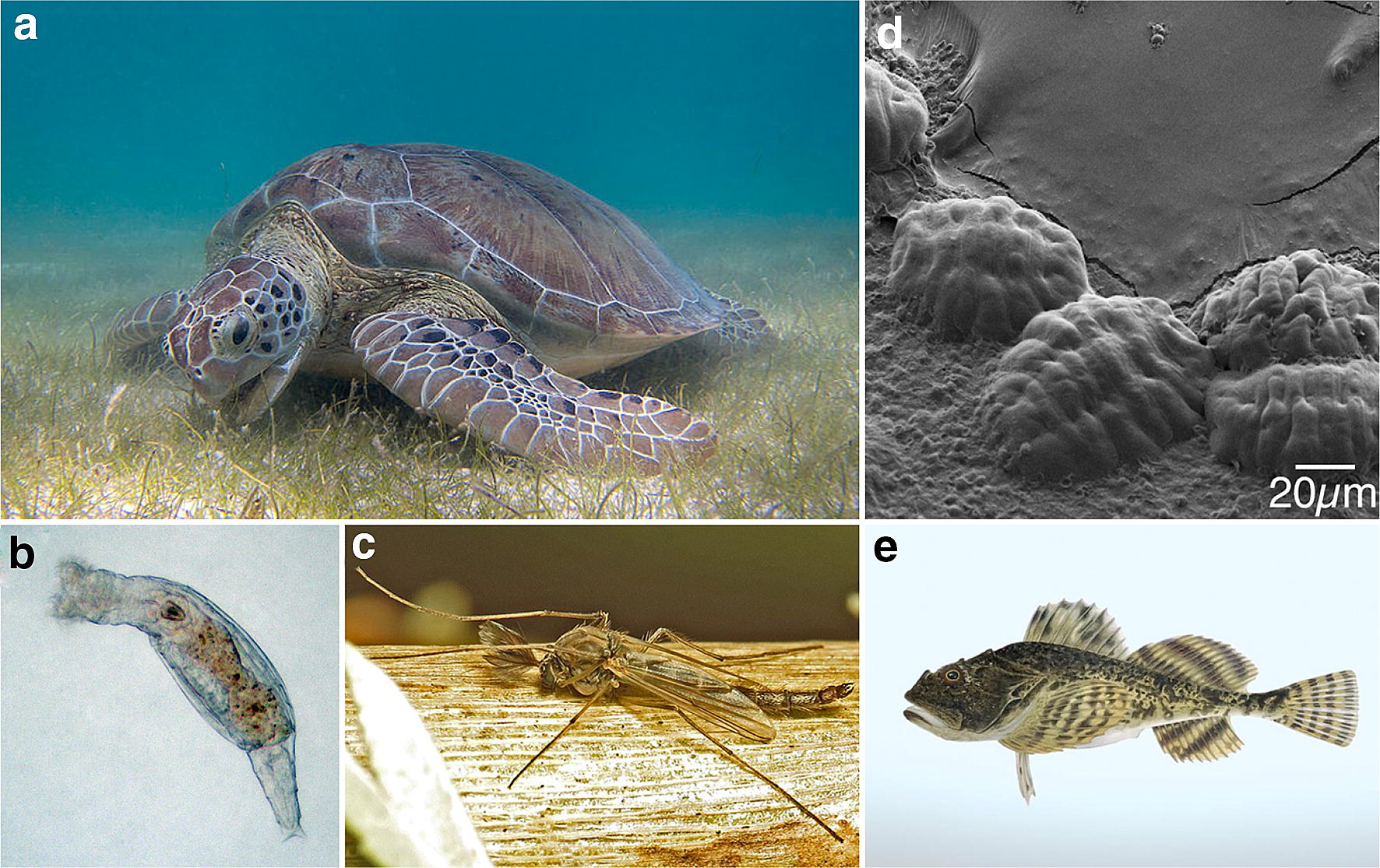
Representative stress-tolerant model organisms. **a** The green sea turtle, *C. mydas*, buries itself in oxygen-poor sediment for up to 2 months for over wintering. Photo credit: P. Lindgren Wikimedia Commons. **b** Bdelloid rotifers survive intense irradiation despite incurring massive DNA damage. Photo credit: Rkiko Wikimedia Commons. **c** Some chironomids, such as *Polypedilum vanderplankii*, have some life-stages that are able to survive extreme desiccation. Photo credit: M. Cooper Wikimedia Commons. **d** SEM image of desiccated tardigrade. When desiccation-tolerant tardigrade dehydrate, they retract their legs and head forming rounded ‘tuns’. Photo credit: Boothby. **e** Depiction of the shorthorn sculpin, am arctic fish that utilizes type I antifreeze proteins to prevent internal ice formation under freezing temperatures. Image credit: Gösta Sundman—Suomen Kalat (Kansalliskirjasto, The National Library of Finland)

The history of life on Earth has been one of adaption and evolution to new and changing environments. Today, in every kingdom of life, we know of both extremophile and extremotolerant organisms that are able to survive environmental stresses that challenge our idea of what the abiotic limits of life are [1]. How animals cope with these environmental extremes and how their ability to do so evolved are questions that have fascinated thousands of researches for centuries, leading to a myriad manuscripts, theses, and books on the subject. Rather than being an exhaustive review on the subject, this review serves as an introduction to five different abiotic stresses as well as extremotolerant animals that have evolved to cope with these stresses. For each stress–animal pair (Fig. 1), the two questions posed above—what cellular mechanisms do animals use to survive these stresses as well as where these abilities may have come from—will be addressed. Given the centuries of research focused on stress tolerance by necessity, many valuable contributions from a number of researchers have not been highlighted. In many cases, these contributions have been covered in works focusing on single forms of stress tolerance, which the reader should seek out if an exhaustive review on a particular subject is needed. The following five vignettes are presented in the hopes that they can serve as a gateway to exploring the mechanistic and evolutionary underpinnings of stress tolerance.

## Hypoxia—buried turtles do not breathe?

**Fig. 2:**
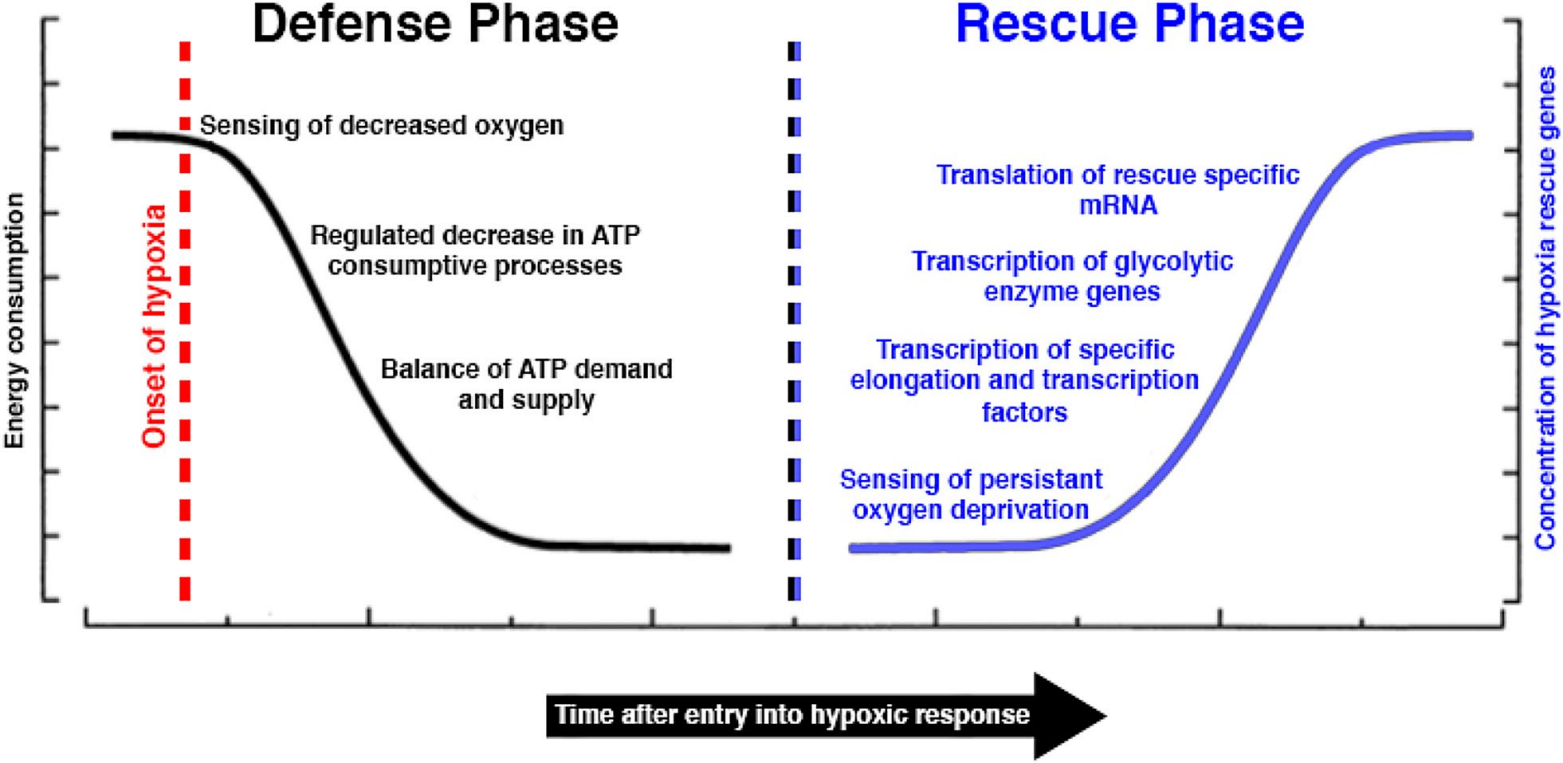
Schematic of defense and rescue phases of hypoxic response. Left: the defense phase of hypoxia tolerance begins with the sensing of low-oxygen conditions. ATP intensive processes, such as membrane ion-pumping and protein synthesis are downregulated. A balance between ATP demand and production is maintained, albeit at decreased levels. Right: the rescue phase of hypoxia tolerance relies on the sensing of sustained oxygen deprivation. Specific upregulation of elongation and transcription factors primes hypoxic cells for recovery. Rescue elongation factors mediate the translation of rescue specific mRNAs, while rescue transcription factors drive the upregulation of rescue specific genes (Adapted from Hochachka et al. [5])

The ability to maintain oxygen homeostasis is vitally important for animals. Oxygen is used by cells to efficiently carry out cellular respiration, the process that produces the energy (ATP) needed by cells to carryout various functions [2]. In humans, hypoxic (low-oxygen) conditions lead to a number of severe physiological consequences including cerebral ischemia (stroke), myocardial ischemia (heart infarction), and tumor growth and metastasis [2]. It is commonly assumed that like humans, most of the animals require steady levels of oxygen to survive. However, it is known that some animals, even vertebrate animals, such as turtles, fish, and frogs, can survive prolonged exposure to hypoxic conditions [3]. For example, during the winter months green sea turtles (*Chelonia mydas*, Fig. 1a) bury themselves in oxygen-poor sediment and can survive up to 2 months under these hypoxic conditions [4]. Through the concerted effort of many researchers, a ‘unified theory’ of hypoxia tolerance has been proposed by Hochachka et al. [5], which proposes that hypoxia tolerance occurs in two phases: the defense and the rescue phases (Fig. 2).

In the defense phase, hypoxic animals balance their ATP demand with ATP supply through a combination of dramatic downregulation of ATP consumptive processes and a modulation of ATP-producing processes [5, 6]. In support of the defense aspect of the theory is the fact that in liver cells from hypoxia-tolerant turtles, there is a 10× reduction of energy turnover [7]. To reduce ATP demand, hypoxia-tolerant organisms are thought to suppress two major cellular processes: protein synthesis and membrane ion-pumping—though other mechanisms clearly contribute to some degree to a decrease in ATP consumption [5, 6]. During low-oxygen conditions, ATP is produced via glycolysis, which is relatively inefficient, producing two ATP molecules per glucose molecule (19 times less efficient than the full potential of a glucose molecule). Somewhat counter-intuitively, rates of glycolysis have often been observed to be reduced during anoxia, in what Hochachka termed the ‘reverse Pasteur effect’ [8]. Storey and colleagues have proposed three major anoxia-induced effects on glycolytic enzymes: phosphorylation and dephosphorylation to alter their activities, binding of enzymes to macromolecules, and allosteric regulation using various metabolites [9, 10]. Reducing ATP demand and supply likely prolongs the length of time animals, such as green turtles, can remain in anoxic conditions by extending the use of stored glycolytic substrate.

During the rescue phase, the expression of key proteins is specifically upregulated in a stepwise fashion. First, under sustained hypoxic conditions, there is a specific upregulation of the translational elongation factor EF1α as well as transcription factor HIF1 [5]. As EF1α accumulates, it mediates the translation of specific rescue mRNAs. The transcription factor HIF1 suppresses expression of genes involved in ATP intensive metabolism, such as enzymes involved in the Kerbs cycle and gluconeogenesis. Meanwhile, genes needed for survival under low ATP-turnover conditions, such as glycolytic enzymes, are upregulated by HIF1 [5]. Ultimately, the combination of defense and rescue mechanisms leads to a lowered, but balanced, ATP supply and demand and the survival of the hypoxia-tolerant animal.

In summing up Hochachka’s theory, the comparative physiologist, Kjell Johansen, likened the approach taken by hypoxia-tolerant organisms to turning down their energy turnover ‘to the pilot light’ level [5]. Kjell’s metaphor is a good one, since clearly, while ATP demand and supply are both lowered, they cannot be extinguished as ATP will eventually be needed to initiate recover from hypoxic conditions. Similarly, while bulk protein production is severally down-regulated during hypoxia, often so quickly that its timeline cannot be accurately assessed [11], completely shutting off protein production is not a viable option, as key ‘rescue’ proteins must be made to eventually mediate a recovery from hypoxic conditions.

One fascinating question with regard to hypoxia tolerance in animals is the question of how or where these traits evolved. Unlike several other stress tolerances, we will address later in this review (e.g., freeze avoidance/tolerance and desiccation tolerance), tolerating hypoxic conditions cannot easily be explained via a single or handful of mechanisms or molecules. Instead hypoxia tolerance is the result of system-wide adjustments in both catabolic and anabolic pathways that span essentially every aspect of cellular physiology.

In thinking about how organisms evolved to tolerate low-oxygen conditions, it is important, and interesting, to remember that for early life, anaerobic microbes, low-oxygen conditions were the norm. It is commonly held that the lack of oxygen in the early Earth’s atmosphere restricted the appearance of animals. However, a recent study provides experimental evidence from sponges, a basal metazoan group, which implicates the last common ancestor of animals as potentially being able to not just survive, but thrive, under hypoxic conditions (0.5–4% of present atmospheric levels of oxygen) [12]. Thus, it is important to consider that tolerance of low-oxygen conditions may have been the norm even for early animal life. With regard to extant animal lineages, namely turtles and fish, that display a heterogeneous distribution of hypoxia tolerance, we must consider whether tolerance to low-oxygen conditions is a trait which was lost and regained in certain species, or a trait that was retained in some species lost in others.

Mapping of hypoxia tolerance to well-established phylogenetic trees points to this trait having evolved independently multiple times within distinct groups of animals [6]. However, a clearer understanding of the commonalities and differences for specific mechanisms and mediators used by these organisms to survive oxygen deprivation is needed to confirm this. If the mediators and mechanisms are identical, then it is less likely that these were convergent events, suggesting that hypoxia tolerance in these lineages was inherited from a basal ancestor (while being lost in sister groups).

## Radiation and rotifers

DNA is the heritable genetic material, which is passed on to our offspring to instruct their development and cellular physiology. Therefore, it seems obvious that stresses, such as irradiation, that result in damage to DNA can be catastrophically bad. However, there are some animals such as tiny invertebrate bdelloid rotifers (Fig. 1b) with the ability to have its genome smashed into tens of thousands of pieces by irradiation. Even after such abuse, the bdelloid rotifer can not only survive, but also can reassemble its genome and produce viable offspring [13, 14].

On Earth radiation is everywhere, albeit at low levels. Annually humans are exposed to ~ 0.0024 Gray (Gy) of ambient background radiation [15]. While life has adapted to these background levels, high levels of radiation (X-rays, γ-rays, and ultraviolet light) can wreak havoc on biological systems through the generation of reactive oxygen species (ROS) [14]. In a cellular context, ROS can lead to the oxidation of essentially any and all types of biological material: DNA, proteins, membranes, and small molecules [16]. Given the damaging effects of irradiation, it is not surprising that most organisms cannot tolerate high levels of exposure. Human cells, for example, will die if exposed to ~ 4 Gy of ionizing radiation [14]. The bdelloid rotifer is able to survive exposure of more than 1000 Gy of radiation [13, 14]. How can such a small, seemingly insignificant animal cope with such stress and where did this ability come from?

When cells are irradiated, they accumulate double-stranded breaks in their DNA, and for a long time it was thought that the massive amounts of DNA damage seen in irradiated cells were what ultimately kills them, and therefore that radiotolerant organisms must protect their DNA from this damage. This makes intuitive sense, however, if this is true then organisms such as rotifers that survive high levels of radiation should have few if any DSBs after exposure. Surprisingly, radiotolerant organisms accumulate DNA lesions at the same rate and to similar levels as those that are radiosensitive [13, 14].

**Fig. 3:**
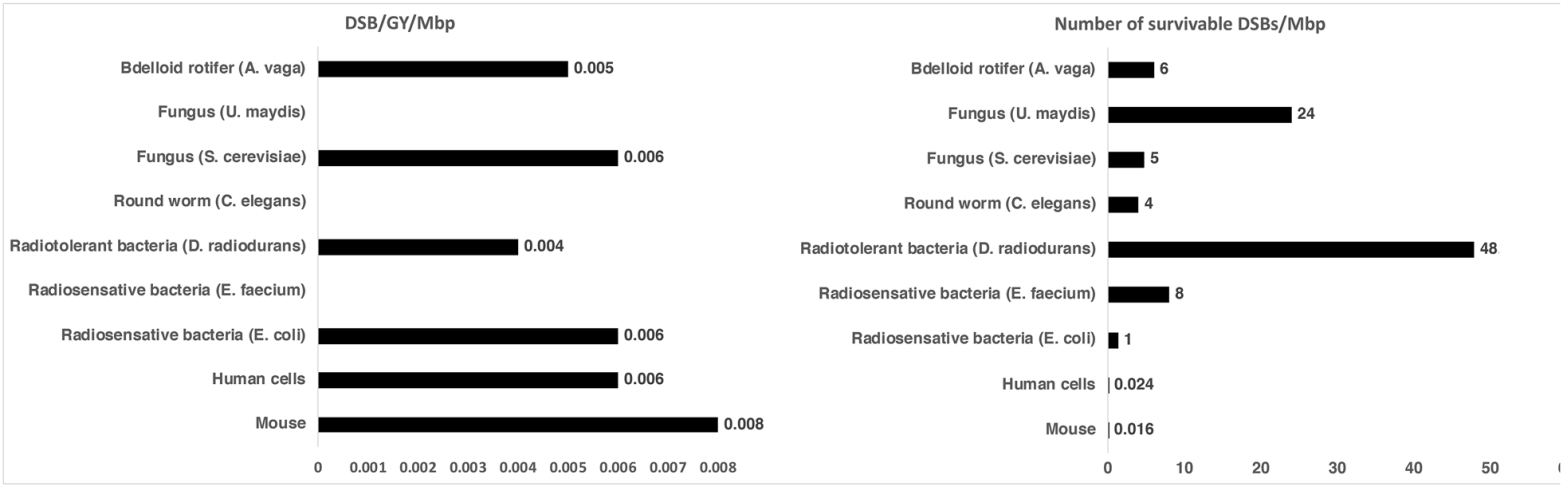
Radiotolerance does not correlate with decreased double-stranded DNA breaks. Left: quantification of DNA double-strand breaks (DSB) per Gray (Gy) of irradiation, normalized for genome size for various radiosensitive and tolerant organisms. Right: number of survivable DSB (normalized to account for genome size) for various radiosensitive and tolerant organisms. *ND* not detected (Adapted from Daly [14])

It turns out that an organism’s ability to survive irradiation does not depend on its ability to protect its genome (Fig. 3), but rather on its ability to protect proteins that will repair its broken genome. One might think that radiotolerant organisms make proteins that are just better at resisting the detrimental effects of irradiation, but this does not appear to be the case. Instead, organisms that survive intense exposure to radiation produce massive amounts of antioxidants, small molecules with which ROS interact with instead of proteins [14].

From an evolutionary standpoint, little is known about how bdelloids or other radiotolerant organisms evolved pathways for antioxidant protection. One interesting point of speculation is that often antioxidants are intermediates in existing biosynthetic pathways. Thus, the disruption of these pathways might lead to an accumulation of antioxidant pathway intermediates. Could radiotolerance have evolved not through the evolution of novel, but rather through the breakdown or disruption of existing metabolic pathways, whose molecule intermediates serve as antioxidants [14]?

## Flies that dry but don’t die

The adage “water is life” seems so obvious. More than 70% of both the Earth and our bodies are made up of water. Water is required for metabolism. Basic cellular components such as lipid membranes and globular proteins require water to maintain their structural integrity. However, despite this, scientists have found organisms spanning every kingdom of life that can survive losing essentially all the hydrating water within their cells.

The first recorded observation of anhydrobiosis or “life without water” is found in a letter from the father of microscopy, Antonie van Leeuwenhoek. In August 1701, van Leeuwenhoek had been observing microscopic animals from rainwater that had collected in a “leaden gutter” in front of his house. By September, due to the “great heat” of the summer, the dirt in the gutter was then “quite dried up” and van Leeuwenhoek took some of this dry dirt and mixed it with rainwater to “see whether living animalcules might be contained in that dry substance.”

What van Leeuwenhoek saw was truly amazing. He described his finding in a 1702 letter:

“… I did not think that any living creature would be present in such a dried-up substance. But I erred in this, for after about one hour I saw at least a hundred of the said animalcules sitting against the glass as well as running along, and swimming.”

The animalcules or “little animals” that van Leeuwenhoek described were most likely rotifers, which we have already discussed in the context of irradiation. Over the past 300 or so years since this first observation, researchers have identified a number of disparate organisms, spanning every kingdom of life, which are able to survive extreme water-loss [17].

*Polypedilum vanderplanki*, a non-biting midge, is the largest anhydrobiotic animal known to science, with its larval form being able to tolerate essentially complete water-loss. These flies live and breed in ephemeral pools of water that form in small (~ 5–9 inches in depth) rocky hollows in Uganda and Northern Nigeria [18–20]. During the rainy season, these pools as well as the flies’ larvae may go through several cycles of hydration and desiccation [18]. The larvae of *P. vanderplanki* have evolved mechanisms that allow them to survive repeatedly being dried out [18–20], but how they survive such insults remained a mystery for over 50 years.

The first clue as to how the larvae of this fly survive desiccation came from the observation that as they dry they accumulate large amounts of the disaccharide trehalose [21]. Trehalose is not unique to *P. vanderplanki*, and is found at very high levels (up to 20% of the dry mass) in a number of desiccation tolerance organisms [22–26] and has been shown to be important for the desiccation tolerance of many of these organisms [27–29].

Two competing, but not mutually exclusive, theories exist which explain how the accumulation of trehalose might help to protect organisms during desiccation [30]. The first theory, called the “Water Replacement Hypothesis” posits that as water is lost, trehalose forms hydrogen bonds with proteins, lipids, and other macromolecules, and as such effectively substitutes for water. A protectant’s ability to effectively mimic hydrogen bonds made by water would have the effect of thermodynamically stabilizing the native conformation of desiccation-sensitive proteins and the structure of membranes. The second theory, known as the “Vitrification Hypothesis” posits that trehalose and other disaccharides (such as sucrose in higher plants) form glass-like matrices as they dry and that macromolecules are physically trapped within. Within this highly viscous matrix, molecular motion is severely reduced, to the point where the motion required for unfolding or structural reengagement is lost. Thus, a vitrified, or glass-like, matrix keeps proteins from denaturing or aggregating together, and membranes from rupturing or coagulating [30].

The identification of high levels of trehalose in dry *P. vanderplanki* larvae [21] prompted researchers to try to answer the question of whether this sugar was acting as a vitrifying or water replacing agent during desiccation of this fly larvae [31].

When Sakurai et al. [31] assayed for the presence of vitrified material in quickly dried (which do not survive desiccation) and slowly dried (which do survive desiccation) larvae, they found that only the slow-dried animals had glassy material present within them, suggesting that there might be some link between the accumulation of vitrified material and the ability to survive desiccation. They went on to test this theory by disrupting the glassy state of vitrified material in slow-dried larvae. First, they heated the larvae up to their glass transition temperature, the temperature at which their glassy accumulations become much more rubbery. The larvae survived heating to, but not beyond their glass transition temperature. Secondly, the researchers increased the humidity of the chambers that the dried larvae were kept in, which plasticized sugar-based glasses, again making them rubberier. They found that increasing the hydration, and therefore rubberiness, of the vitrified larvae lowered its glass transition temperature, until this value fell below ambient laboratory temperatures and the glass disappeared. Larvae survived desiccation, even with humidity plasticized glasses, but once those glasses were no longer stabile at room temperature, survival dropped quickly to 0%. In other words, only larvae that have vitreous material survive desiccation and if you disrupt the glassiness of that material in otherwise viable specimens, they can no longer survive [31]. Therefore, it looks like trehalose is probably working through vitrification.

**Fig. 4:**
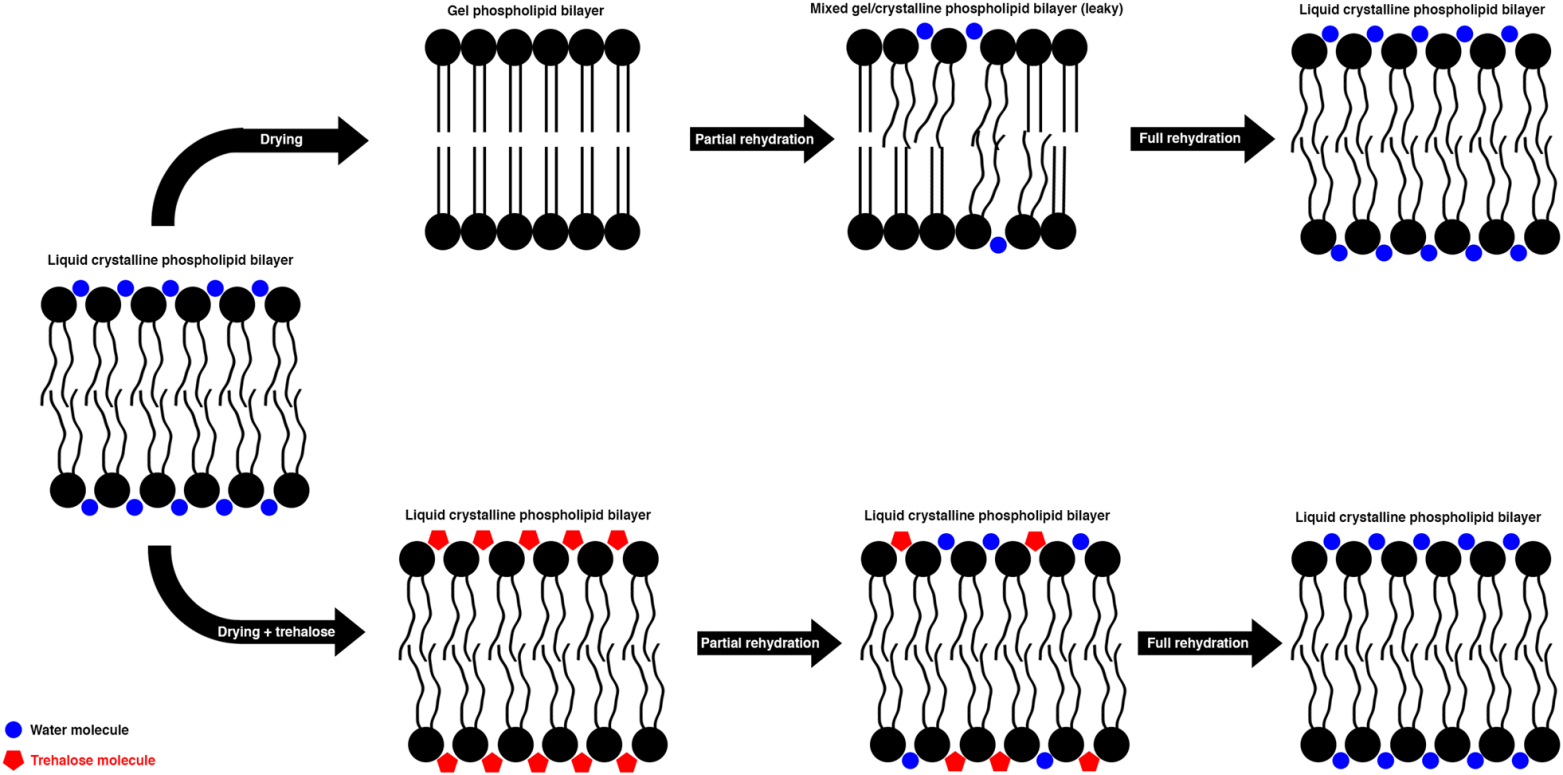
Schematic representation of trehalose’s proposed protective mechanism. Hydrated phospholipid membranes exist in a liquid crystalline state. Under normal desiccation conditions (top), dry membranes transit to a gel state. Rehydration causes transient leakiness as membranes in a gel state transit back to a liquid crystalline state. When desiccated in the presence of trehalose, the disaccharide maintain the spacing of phospholipid head groups (either via water replacement or vitrification—or both), allowing membranes to maintain their liquid crystalline state. (Adapted from Crowe et al. [78])

However, the researchers looked at whether or not trehalose might also be forming hydrogen bonds with cellular macromolecules, such that the sugar acts to replace water. They found evidence of phospholipid–sugar hydrogen bonding in slowly, but not quickly dried larvae, and furthermore that these interactions stabilize membranes in a liquid crystalline state. This is important because when hydrating water is lost, membranes undergo a shift from being in a liquid state to a gel state (Fig. 4). This can have a number of deleterious effects, including separation of membrane components, fusion of membranes, and the presence of mismatched gel and liquid portions of membranes, which generates leakage [32–34]. Therefore, the observation that sugar–phospholipid hydrogen bonding is potentially preventing this deleterious shift from liquid to gel states in membranes is good evidence that trehalose maybe acting to replace water in *P. vanderplanki*.

In summary, the midge *P. vanderplanki* is thought to survive desiccation via the accumulation of high levels of trehalose. Trehalose likely acts both to vitrify the inside of desiccation-tolerant animal cells reducing the levels of molecular motion required for protein denaturation as well as forming hydrogen bonds with phospholipids, thus replacing water, and keeping membranes from leaking during dehydration/rehydration cycles [31].

A recent analysis shows that trehalose biosynthetic pathways are present in bacteria, archaea, plants, fungi, and animals [35]. While bacteria and archaea have evolved five different biosynthetic pathways to make trehalose, animals, plants, and fungi have only one known trehalose biosynthetic pathway, which is called the trehalose-6-phophate synthase (TPS)—trehalose-phosphatase (TPP) pathway. In general, the evolution of these pathways has occurred mostly in parallel, but there has been speculation that lateral (horizontal) gene transfer may have occurs several times [35, 36].

It is interesting to note that desiccation tolerance and several other abiotic stress tolerances, such as radiotolerance (see above) and thermotolerance (see below) might be mechanistically and evolutionary linked [13, 37]. Dramatically increased levels of ROS and DNA damage are hallmarks of both irradiation and desiccation [13]. Furthermore, many desiccation-tolerant organisms are also radiotolerant [13]. The question of whether organisms that survive both these stresses use overlapping mechanisms to do so is of immense interest and importance. Likewise, vitrification, as in desiccation tolerance, has been linked to the ability of some organisms to survive high temperatures (see below). Identifying the mechanistic commonalities and differences between different forms of stress tolerance will ultimately lead to a better understanding of how these different stress tolerances arose.

## Tardigrades and thermotolerance

High temperatures can wreak havoc on organisms as well as their macromolecules that have evolved to function under lower thermal conditions. At high temperature proteins unfold and form nonfunctional aggregates. Similarly, membranes can fuse and rupture. While there are organisms that have specifically evolved to thrive under high temperatures, such as bacteria and archaea living in hot springs, there are also organisms that have evolved not to thrive, but to tolerate conditions well above their optimal temperatures.

**Fig. 5:**
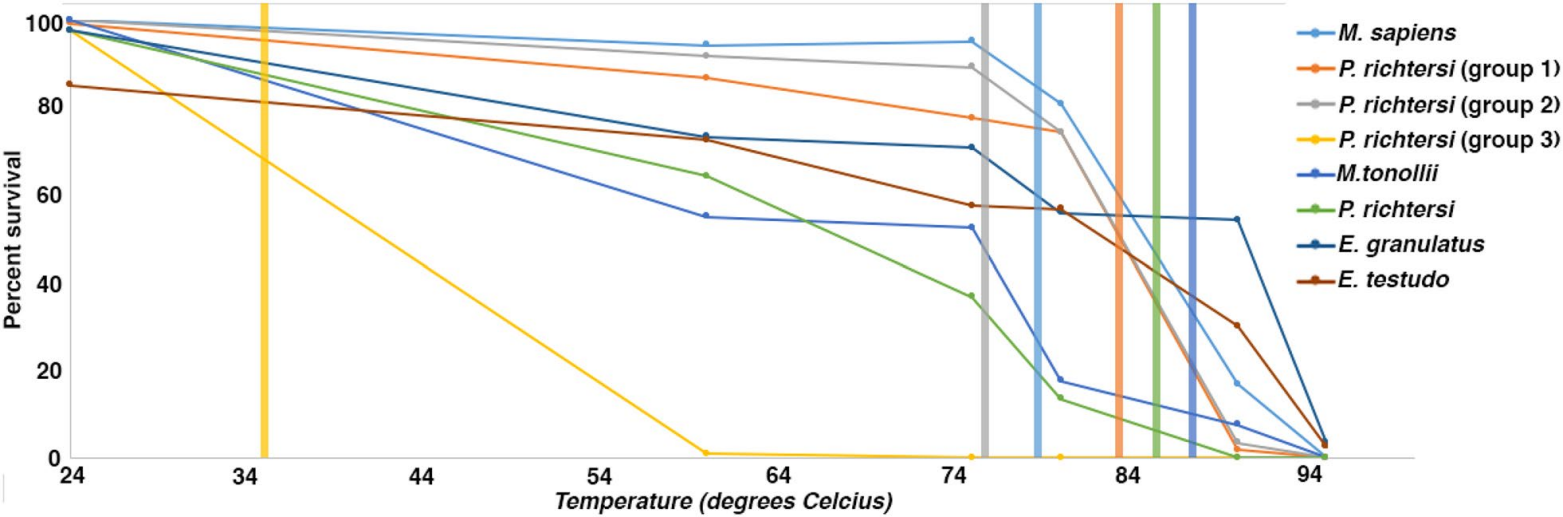
Glass transition temperature correlates with survival of high temperatures. Graph showing the percent survival of desiccated tardigrades after heating. Vertical lines indicate experimentally measured glass transition temperatures for different tardigrade species (Data from Hengherr et al. [37])

One such animal is the tardigrade, more commonly known as the water bear (Fig. 1d). Tardigrades are a group of microscopic animals renowned for their ability to survive a number of environmental extremes including desiccation [38], freezing [39], intense radiation [40], extreme pressures [41], and temperatures up for 151 °C [42]. Interestingly, thermotolerance in tardigrades appears linked to their ability to desiccate, with tardigrades, like many other desiccation-tolerant organisms, tolerating much higher temperatures when dry compared to hydrated [37, 43]. An interesting clue to what allows tardigrades to tolerate high temperatures when desiccated comes from Hengherr et al. [37] who found that tardigrades vitrify when dried (like the fly *P. vanderplanki* in the previous section on desiccation) and that the vitreous state of these animals correlates with their ability to tolerate high temperature. In this vitreous state, many anhydrobiotic tardigrade species survive temperatures of up ~ 100 °C for at least an hour with some species surviving even greater temperatures [37]. Furthermore, disruption of the vitrified state at high temperatures correlates with severe decreases in the survival of tardigrades (Fig. 5; [37]). These findings were confirmed by a later study, which also identified tardigrade-specific intrinsically disordered proteins as being linked to vitrification and survival [44].

When biological material, such as proteins, DNA, and membranes, are heated, they denature and aggregate, fragment, or rupture. As discussed above in the section on desiccation, materials that vitrify are thought to help prevent these detrimental processes by physically trapping biological molecules in a glass-like matrix. Trapped in this matrix, proteins cannot denature, DNA is kept annealed and unfragmenting, and the integrity of membranes is preserved [30, 45].

As mentioned in the preceding section on desiccation, vitrification in the context of stress tolerance has most often been attributed to high levels of the disaccharide trehalose. However, based on biochemical analysis, it does not appear that tardigrades accumulate high levels of trehalose [46–48], and there is speculation that they may not possess the biosynthetic pathways to produce this sugar for themselves [49]. Thus, some other molecule(s), besides trehalose, might be response for producing the vitrified states in tardigrades that correlate with tolerance to high temperatures. What might this other molecule(s) be? Another sugar, besides trehalose, is a possibility. But intriguingly, there is speculation that intrinsically disordered proteins (IDPs) might mediate vitrification [31]. IDPs are a class of enigmatic proteins that lack a stabile 3-dimensional structure and there are several families of disparate IDPs from a broad range of organisms, including tardigrades, which have known or suspected links to stress tolerance [50–53]. Additionally, it is known that denatured globular proteins (essentially IDPs) vitrify upon desiccation, and that the addition of IDPs to trehalose strengthens the resulting glass [54, 55]. Could endogenously disordered proteins form glasses on their own? One study in tardigrades has linked the ability of these animals to survive desiccation to the production of IDPs [44]. However, further experiments will be needed to confirm the widespread ability of IDPs to form glasses on their own. Despite what will be found in other organisms, the ability of tardigrades to use protein-based glasses to tolerate desiccation and high temperatures represents an elegant example of how evolution can converge on a similar mechanism (vitrification) via two distinct mediators (a sugar versus a protein) [44].

As we touched on in our section on desiccation, several forms of stress tolerance are suspected to be mechanistically and evolutionarily linked. Along with radiotolerance, thermotolerance may also be mechanistically linked to desiccation tolerance. Many thermotolerant animals are only survive high temperatures when dehydrated and, in some cases, functional molecule(s) (e.g., trehalose) may be the same. Elucidating the functional mediators of these stresses will not only tell us a great deal about how organisms evolved resistance to different abiotic extremes, but also will provide avenues for pursuing real world applications, such as stabilizing and extending the shelf life of pharmaceuticals and engineering stress tolerant crops.

## Why fish in the arctic do not freeze?

While we have seen that some animals, such as desiccated tardigrades, survive high temperatures, there are other organisms that do the opposite—surviving temperatures well below conditions at which they should freeze. The shorthorn sculpin (Fig. 1e), *Myoxocephalus scorpius*, a fish found living near the ocean floor of the North Atlantic into the Arctic Ocean, is one such cold-tolerant organism. The shorthorn sculpin, besides apparently being good bait for lobster traps, is not commercially important or endangered [56]. However, this is a fish that arguably every biologist should know about, because it does something very interesting, or rather what it does *not* do is interesting—it does not freeze [57–59].

In the more northern ranges of the shorthorn sculpin, water temperatures can reach close to the freezing point of salt water (− 2 °C), below the point at which most marine fish freeze (− 0.8 °C) [60]. However, unlike most fish at these temperatures, the shorthorn sculpin does not freeze. How does this otherwise quite unremarkable fish avoid freezing at these temperatures and how did it evolve this ability?

Before we dive into understanding how the shorthorn sculpin survives these freezing conditions, we should consider what happens to organisms and their cells when they freeze. The most obvious change, besides temperature, is the amount of free water to carryout metabolism with decreases. When the water inside an organism’s cells freezes, there is no long an aqueous medium in which metabolic reactions can occur. Along with this, reducing the availability of free water results in a hypertonic solution, which represents an enormous homeostatic strain. Additionally, ice crystals that form during freezing will expand, puncturing and disrupting the integrity of cellular membranes and tissues, in addition to destroying proteins and nucleic acids [1]. Thus, it is not surprising that there are a number of diverse organisms that are either freeze avoidant or freeze tolerant [1].

So, how does the shorthorn sculpin avoid having its cells and fluids freeze, and thus avoid the detrimental effects associated with internal ice formation? The shorthorn sculpin, and many other freeze-tolerant organisms, rely on antifreeze proteins (AFPs) [60]. There are several different classes of antifreeze proteins, which are all thought to work through a similar mechanism—ice growth inhibition [60]. As water begins to freeze, small ice crystals form, which act as nucleation points facilitating the freezing and crystallization of surrounding water. AFPs work by adsorbed to the surface of these crystals while they are still small. At the surface of ice crystals, it is thought that AFPs essentially act as shields, blocking the growth of small ice crystals into larger damaging ones [60, 61].

The sculpin relies on a particular class known as Type I antifreeze proteins [58, 62]. The shorthorn sculpin is not the only fish that possesses Type I AFPs, and there are of course differences between the structure and sequence of Type I AFPs found in different species of fish [58, 63].

**Fig. 6:**
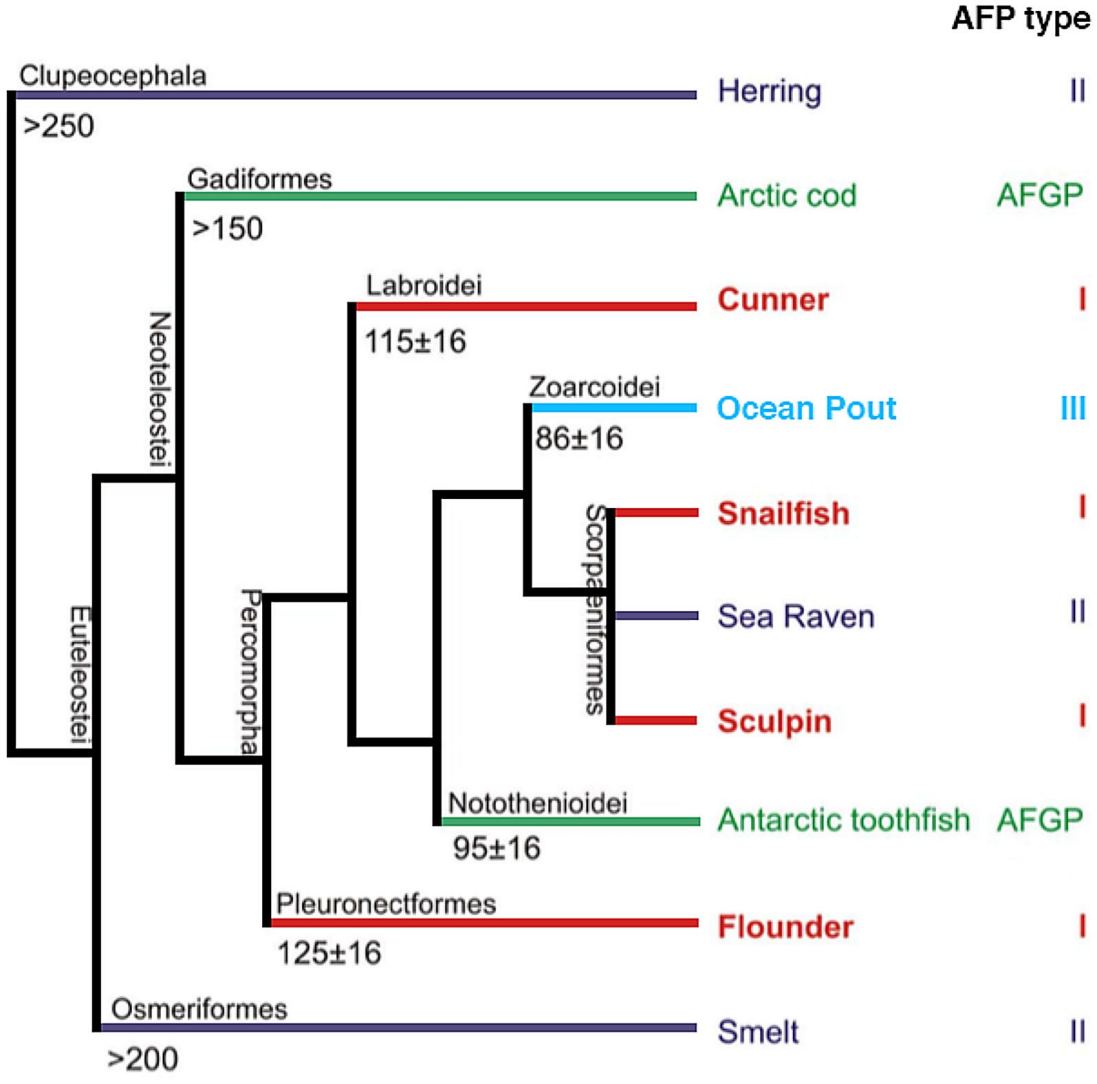
Antifreeze proteins show a distinctly disperse distribution among fish lineages. Phylogenetic tree showing distribution of Type I (red), Type II (purple), Type III (blue), and AFGP (green) antifreeze proteins among fish lineages (Adapted from Graham et al. [59])

Interestingly, Type I AFPs show a markedly dispersed distribution among different fish linages, having been found in 4 superfamilies (Cunners, Snailfish, Flounder, and Sculpin) across 3 different orders of fish (Fig. 6; [58, 59, 63]). Type I AFPs are not the only class of AFP that shows dispersed distribution and fish within the same order are known to possess different classes of AFPs (Fig. 6; [59]). How did such a strange distribution of AFPs arise?

Around 260 million years ago, the Karoo Ice Age ended and the Earth became relatively warm until the start of the current ice age, ~ 2.6 million years ago [64]. Many groups of fish, which today rely on Type I AFPs to survive freezing temperatures, diverged during this interglacial period [65–67]. While, it is possible that AFPs are an adaptation conserved during this interglacial period, another, more likely possibility, is that AFPs evolved after this interglacial period, after the divergence of many AFP-reliant fish [59]. If true, the identification of Type I AFPs in disparate superfamilies and orders of arctic fish would represent multiple convergent evolutionary events.

If Type I AFPs arose multiple times in diverse fish species, what precursors did they arise from? There are several possibilities that have been proposed. Graham et al. [59], examined low complexity alignments between Type I AFPs and teleost fish sequences from multiple GenBank databases. They found that many of the resulting alignments were to microsatellites with repeated trinucleotide stretches of GCT or GCA, which could potentially encode long runs of alanine residues, a hallmark of Type I AFPs. Thus, microsatellites might serve as a common origin of diverse Type I AFPs [59]. An earlier study found that some Type I AFPs had runs of residues with significant alignments, both at the nucleotide and protein level, to egg shell proteins and Type II keratin from snailfish [63]. Both these egg shell and keratin proteins have long runs of glycine residues, which the authors postulate could undergo a frameshift to generate a protein with high homology to known snailfish Type I AFPs [63]. In addition to frameshifts in glycine-rich proteins, frameshifts in proteins rich in residues besides glycine might also result in Type I AFP-like proteins with long runs of alanine residues (a hallmark of Type I AFPs) [59]. A final possibility is that short segments of DNA encoding alanine residues were duplicated, resulting in long stretches of alanine residues [59].

## Conclusions

The preceding vignettes of this chapter are by no means meant to be an exhaustive review of extremophile or extremotolerant animals and mechanisms. But what do they tell us about the cellular mechanisms and evolution of stress tolerance?

## Mechanisms of protection against extreme environmental stresses—preventing versus fixing damage

Typically, biological material will be damaged when exposed to extreme abiotic stresses. Answering question of whether this damage is prevented, efficiently repaired, or both, is important for understanding the cellular mechanisms of extremotolerant organisms. In the preceding chapter, we have seen examples of each of these possibilities. When exposed to intense irradiation, rotifers incur large numbers of DNA damage, which they must efficiently repair to survive [13, 14]. At the same time, rotifers have mechanisms, likely elevated levels of antioxidants, that allow them to prevent damage to the proteins that will ultimately repair this DNA damage [13, 14]. Desiccation is similar, where the midge *P. vanderplanki* utilizes the disaccharide trehalose, which is thought to prevent the denaturing and aggregation of proteins as well as the disruption of membranes through a combination of vitrification and water replacement [30, 31]. Similar to irradiation, desiccation is known to induce a high degree of DNA damage, even in desiccation-tolerant organisms, and this damage is efficiently repaired only after rehydration [68–71]. Thus, we can see from only a few examples that mechanism of extreme environmental tolerance can work at both the level of protection and repair, and often coordination of these mechanisms essential for survival.

## Specific and general stress responsive mechanisms

Many of the organisms we have discussed in the preceding chapter, e.g., tardigrades and rotifers, are polyextremotolerant. That is, they can survive more than one extreme environmental stress. One question that arises from this observation is whether or not polyextremophile or polyextremotolerant organisms use overlapping, distinct, or a combination of overlapping and distinct mechanisms to survive different types of stress.

The answer to this question is further complicated by the fact that some stresses illicit similar detrimental effects, while others do not. For example, both desiccation and irradiation lead to extensive DNA damage [13, 14, 68–71], whereas this is not such a concern for hypoxia. Freezing and desiccation can both cause the disruption of membranes, but through different physiological processes, ice crystal formation and expansion for freezing and liquid–gel phase transitions for desiccation [34, 72].

Studies addressing the mechanistic connection or ‘cross tolerance’ between different stresses have suggested that there are mechanistic links between different tolerances. For example, the goldenrod gall fly (*Eurosta solidaginis*), was shown to be better able to survive freezing after being exposed to mild desiccation [73]. However, accumulated work has shown that in yeast, the mechanisms of stress tolerance (and cross tolerance) depend on, and are specific to, exposure to different types of stress [74].

In thinking about cross tolerance, it is important to remember that resistance to the same environmental stress has almost certainly arisen multiple independent times in different animal linages [6, 59]. Thus, while in some lineages cross tolerance may not be observed, this observation does not rule out the possibility of independent evolution of cross tolerant mechanisms in different linages. Ultimately, further elucidation of functional mediators of stress tolerance and their mechanisms of action will provide a more complete picture about mechanistic and evolutionary links between different forms of stress resistance.

## Evolutionary paths to stress tolerance

How novel traits arise is a major question in evolutionary biology. With regard to stress tolerance, not surprisingly, we have seen that evolutionary novelty can arise through a variety of means. Diverse families of APFs, while functionally similar, likely arose from different ancestral proteins, and even AFPs within the same family may have arisen in different lineages via different means (e.g., frameshifts *versus* serial duplications) [59]. Radiation tolerance may have come about not through the evolution of novel biosynthetic pathways, but through a breakdown or disruption of existing pathways, leading to the accumulation of antioxidant intermediates [14]. Horizontal gene transfer, genomic incorporation of DNA from other organisms, has been implicated in the acquisition of novel stress tolerant traits [35, 36, 75–77]. In other cases, hypoxia for example, evolution of regulatory control over existing metabolic and physiological processes has given rise to tolerance [6]. Thus, we can see that are myriad ways in which stress tolerances can and have arisen.

Currently, there is little evidence to suggest that there are hard-and-fast rules regarding functional or evolutionary mechanisms for different stress tolerances. While mechanistic overlap is speculated to exist for some forms of stress tolerance, for other forms there is little indication of such overlap. Similarly, the evolutionary routes for a species or linage acquiring tolerance(s) to stress appear to be many and varied. Further work will be needed to elucidate the mediators of diverse stress tolerance and their mechanism(s) of action. Doing so will allow for robust evolutionary conclusions to be drawn and promise to contribute to real world applications, such as the engineering to stress tolerance crops and the development of novel methods for stabilizing biomedically relevant material.

## Data Availability

Not applicable.

## Abbreviations

AFGP: antifreeze glycoprotein
AFP: antifreeze protein
DSB: double strand break
Gy: Gray
IDP: intrinsically disordered protein
ROS: reactive oxygen species
TPP: trehalose-phosphatase
TPS: trehalose-6-phosphate synthase
